# Implementing parallel spreadsheet models for health policy decisions: The impact of unintentional errors on model projections

**DOI:** 10.1371/journal.pone.0194916

**Published:** 2018-03-23

**Authors:** Stephanie L. Bailey, Rose S. Bono, Denis Nash, April D. Kimmel

**Affiliations:** 1 Department of Health Behavior and Policy, Virginia Commonwealth University, Richmond, Virginia, United States of America; 2 Physics Department, University of California–Santa Cruz, Santa Cruz, California, United States of America; 3 Department of Epidemiology and Biostatistics, City University of New York, New York, United States of America; Public Health Agency of Canada, CANADA

## Abstract

**Background:**

Spreadsheet software is increasingly used to implement systems science models informing health policy decisions, both in academia and in practice where technical capacity may be limited. However, spreadsheet models are prone to unintentional errors that may not always be identified using standard error-checking techniques. Our objective was to illustrate, through a methodologic case study analysis, the impact of unintentional errors on model projections by implementing parallel model versions.

**Methods:**

We leveraged a real-world need to revise an existing spreadsheet model designed to inform HIV policy. We developed three parallel versions of a previously validated spreadsheet-based model; versions differed by the spreadsheet cell-referencing approach (named single cells; column/row references; named matrices). For each version, we implemented three model revisions (re-entry into care; guideline-concordant treatment initiation; immediate treatment initiation). After standard error-checking, we identified unintentional errors by comparing model output across the three versions. Concordant model output across all versions was considered error-free. We calculated the impact of unintentional errors as the percentage difference in model projections between model versions with and without unintentional errors, using +/-5% difference to define a material error.

**Results:**

We identified 58 original and 4,331 propagated unintentional errors across all model versions and revisions. Over 40% (24/58) of original unintentional errors occurred in the column/row reference model version; most (23/24) were due to incorrect cell references. Overall, >20% of model spreadsheet cells had material unintentional errors. When examining error impact along the HIV care continuum, the percentage difference between versions with and without unintentional errors ranged from +3% to +16% (named single cells), +26% to +76% (column/row reference), and 0% (named matrices).

**Conclusions:**

Standard error-checking techniques may not identify all errors in spreadsheet-based models. Comparing parallel model versions can aid in identifying unintentional errors and promoting reliable model projections, particularly when resources are limited.

## Introduction

Systems science approaches are increasingly used to inform health policy decisions and the allocation of scarce resources [1]. While a variety of frameworks exist to represent a system, model implementation is often complex and unclear, hindering acceptance of model results.

To promote the uptake of model findings into practice, the health-related literature and international guidance on best modeling practices emphasize two model characteristics: accessibility (i.e., ease of model use and reliance on widely available software) and transparency (i.e., clarity in the methods used to develop and implement models) [2–7]. Model implementation using spreadsheet software can improve both. Spreadsheet software allows for clear presentation of a model’s structure or health states, values for the data used to populate the model, and mathematical relationships between the health states and data. Spreadsheet model calculations are immediately accessible, as is the impact of changes in the input data on the model’s projected outcomes, all in real time to reflect local needs and with minimal training. The inherent transparency of spreadsheet models thus promotes model credibility, which is valued highly by decision makers [7].

Spreadsheet-based models, while transparent and accessible, are cumbersome and prone to implementation error [8]. These errors can have profound policy implications [9–11], particularly in contexts with constrained resources and limited technical capacity. While best practice guidelines on error identification approaches do exist (e.g., assessment of model performance, double-programming) [4, 7], practical, straightforward, and evidence-based guidance is limited on approaches to identify errors beyond standard error identification techniques and for settings with limited resources. Capitalizing on a real-world need to make policy-relevant updates to an existing spreadsheet-based model, the HIV Policy Model [12, 13], we aimed to illustrate, through a methodologic case study analysis, the impact of unintentional errors on model projections by implementing parallel model versions.

## Materials and methods

### Study design

We conducted a methodologic case study analysis in order to demonstrate the feasibility of the proposed method—i.e., implementation of parallel model structures—in identifying unintentional errors for spreadsheet models developed and updated in real-world settings. The analysis was designed to demonstrate the method’s practical potential by illustrating the substantial impact that unintentional errors can have in informing policy decisions.

We emphasize that this methodologic demonstration was intended to bridge the gap between academia and practice by addressing the need for practical, timely solutions for avoiding unintentional model errors in real-world settings [2]. Modeling guidelines recommend, among other well-established model verification techniques, use of double-programming, in which multiple trained programmers independently implement models [4]. However, implementation of this approach may not be feasible due to lack of technical capacity, time, or financial resources [7]. In the context of increasing interest from decision makers from a variety of resource-limited settings (such as state health departments and ministries of health) in generating evidence to inform their decisions [14, 15], practical new approaches are necessary. Broadly, this methodologic demonstration involves mimicking real-world conditions (i.e., a single programmer) and demonstrating that during model revision, unintentional errors can occur, can be identified, and have the potential for substantial policy impact. As importantly, we demonstrate that this practical, straightforward solution can be implemented and used to identify errors that might otherwise go unnoticed.

### Existing spreadsheet model: The HIV Policy Model

The HIV Policy Model is a multi-cohort, state-transition model of treated and untreated HIV disease, originally developed for Haitian decision makers to examine the number of deaths averted by earlier antiretroviral therapy (ART) initiation and scale-up. The model employs a conceptually simple structure and is implemented using widely available spreadsheet software (Microsoft Excel, Redmond, WA, USA). The original deterministic model includes 13 main health states, subdivided to characterize the nuances of disease progression and care engagement for a total of 48 health states. These health states are captured in approximately 1,100 spreadsheet cells over a 15-year policy-relevant time horizon. Successive, hypothetical cohorts of HIV-positive individuals enter the model annually and progress through three mutually exclusive, collectively exhaustive stages of untreated disease that are defined according to immunologic status (i.e., CD4 cell count). The stages of untreated disease are: Asymptomatic (corresponding to CD4 count >350 cells/µL), Intermediate (CD4 count 200–350 cells/µL), and AIDS (CD4 count <200 cells/µL). Within each disease stage, fractions of a cohort remain out of care, enter pre-treatment care, initiate ART, become lost to follow-up, or die. Transition probabilities are derived from local patient-level clinical data from three observational cohorts and a randomized trial of early versus delayed ART [16–22], conducted over more than twenty years by the Haitian Study Group for Kaposi’s Sarcoma and Opportunistic Infections clinic that provides comprehensive HIV testing, treatment, and related services in Port-au-Prince, Haiti. The model’s performance is assessed systematically and iteratively through face validity checks, examining model behavior (e.g., logic checks, identification of implementation errors), testing internal validity, and corroborating model projections using external data sources [4, 7, 13]. Additional details on the model structure, data used to parameterize the model, and model calibration and validation process, as well as findings from policy analyses, are available elsewhere [12, 13].

### Parallel model versions

We developed the initial HIV Policy Model to be compact, with all transition probabilities to a given health state characterized in a single cell formula and, following best practices, relying primarily on the use of cell names [23]; we refer to this version as Single Name. We compared two additional versions: Single Cell retained the same structure and format but used column/row cell references (e.g., A1), instead of cell names. Matrix used transition probability matrices, with health state transitions occurring across multiple cells and relying on cell names (for transition probability matrices) and cell references (for matrix multiplication). Prior to revisions described below, Matrix had approximately 37,000 cells versus the 1,100 in the other versions. However, for ease in error identification and impact calculation across model versions, we also presented Matrix output in the same compact structure as Single Name and Single Cell.

### Model revisions

**Fig 1:**
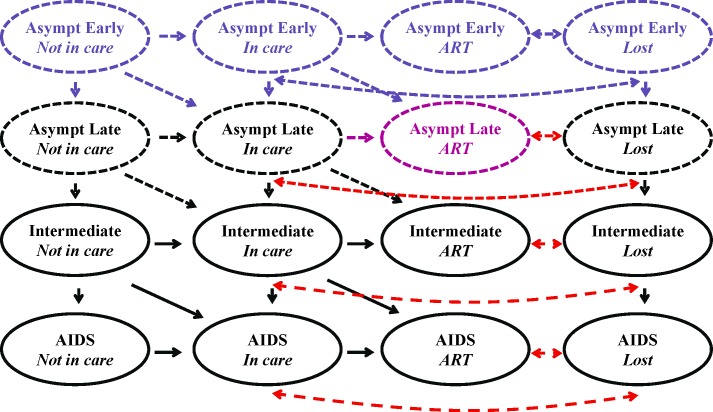
Schematic of revisions to the HIV Policy Model. Shown is a simplified schematic of the HIV Policy Model. Each oval represents a main health state and the arrows represent transitions between health states. Dotted lines reflect new health states or transition probabilities, while colors indicate the implemented model revision. Red represents the Re-entry into Care revision, purple the Universal ART revision, and blue the Guideline-based ART revision. Death from AIDS- or non-AIDS-related causes is not shown.

Across all versions, we assessed three policy-relevant revisions to evaluate unintentional errors and their impact on model projections (Fig 1). The first revision, Re-entry into Care, reflected realistic clinical care engagement practices by incorporating new transitions from existing health states: patients lost to follow-up in the Intermediate or AIDS health states could return to care. The second, Universal ART, allowed for immediate initiation of ART upon diagnosis, regardless of stage of disease progression and in line with the global debate then surrounding guidelines for antiretroviral initiation in low-income settings [24–26]. The third, Guideline-based ART, incorporated new health states allowing for antiretroviral initiation in line with new guidelines at the time of model revision [27]; this accommodated antiretroviral initiation earlier in the course of disease than the original model but later than the Universal ART revision. International guidelines for ART initiation changed twice during model development, requiring model revisions to remain up-to-date. One programmer implemented all revisions. For each model revision, fidelity to the revision specification was enhanced via written documentation of the planned revision, detailed graphical representations of the planned revisions, comprehensive logs outlining all model updates, and comparison of the revised model to the revision plan.

### Analytic approach and policy impact

**Fig 2:**
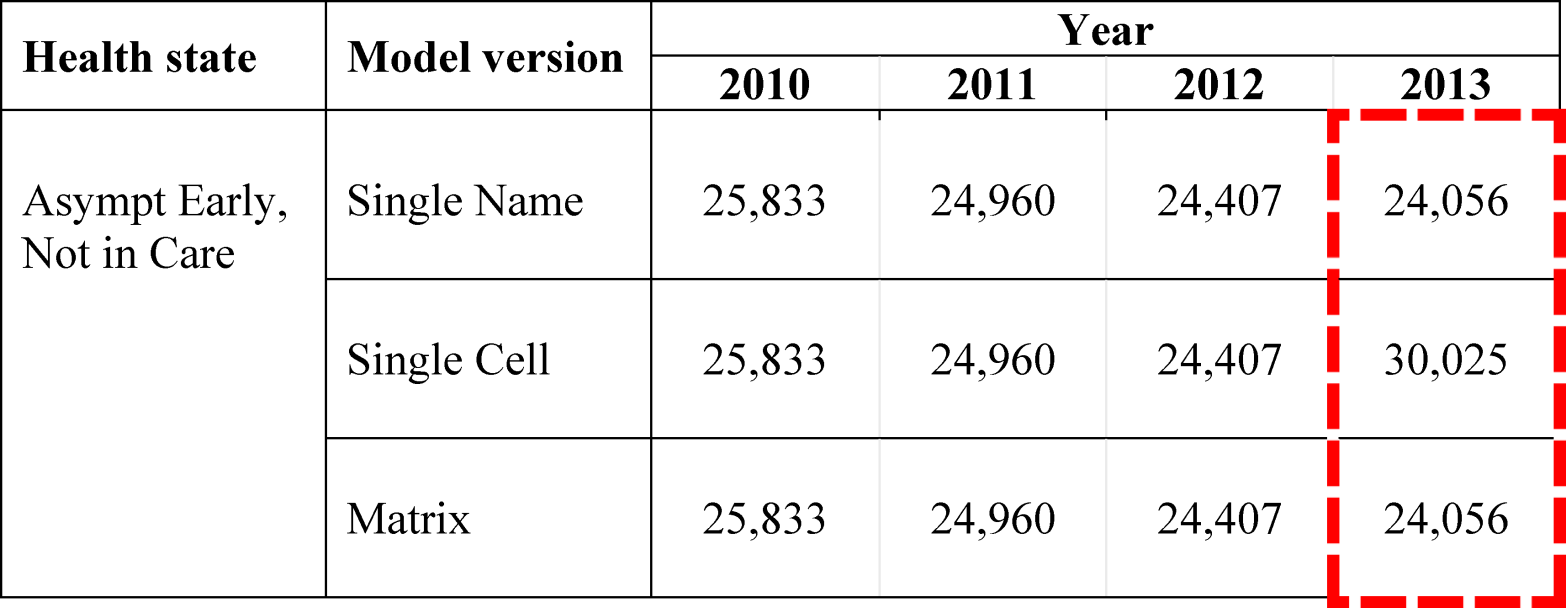
Example of unintentional errors detected by comparing model projections. This figure is a snapshot of projections from all three model versions (Single Name, Single Cell, Matrix) for the Guideline-based ART revision. Each cell in this snapshot shows the number of people living with HIV projected to be in a given health state (Asymptomatic Early stage of disease, Not engaged in care) between 2010 and 2013, after standard debugging. The figure shows that model output for this health state is identical across the three model versions for 2010, 2011, and 2012, indicating no unintentional implementation errors. However, in 2013, the projected output for this health state differs across the three model versions (Single Name: 24,056, Single Cell: 30,025, Matrix: 24,056). In this example of an unintentional error, the principle error was due to an incorrect cell reference in the Single Cell model version, was propagated over an additional 10 health states, and took 10 minutes to identify and correct. Full model projections for the Guideline-based ART revision with unintentional errors are available in tabs Model (SN), Model (SC), and Model (Matrix) in S5 File.

To identify errors, we implemented a given revision across all three model versions, conducted standard debugging techniques following best practices for systems modeling [4], and compared output for all three model versions simultaneously to identify remaining unintentional errors. Standard error identification approaches included: face validity checks (e.g., simple checks of output reasonableness), negative output tests (i.e., functionality checks to confirm that the model does not project negative output), extreme value analysis (e.g., mortality risk for a given value at either 0 or 1), alternative assumptions about the model’s initial cohort distribution across health states (e.g., the initial cohort is in a single health state), and tracking of the size of a single cohort over time [4, 7, 28]. To detect unintentional errors, we identified discrepancies in output across model versions, assuming that models free of unintentional errors should project identical output. Specifically, for each revision, an error was detected when the difference in the projected number for a given spreadsheet cell between two model versions was not zero (Eqs 1–3):

$$n_{c,\;SN}-n_{c,\;SC}\neq 0$$ (1)

$$n_{c,\;\;SN}-n_{c,\;M}\neq 0$$ (2)

$$n_{c,\;SC}-n_{c,\;M}\neq 0$$ (3)

where *n* is the projected number in spreadsheet cell *c* for a given model (*SN*: Single Name, *SC*: Single Cell, *M*: Matrix). This process is depicted visually in Fig 2 and is shown in tabs Diff (SN-SC), Diff (SN-Matrix), and Diff (SC-Matrix) in S1 File, S3 File and S5 File. The identification process for unintentional errors was complete when we corrected all identified unintentional errors and found no discrepancies in output across versions. That is, for each corresponding cell across model versions:

$$n_{c,\;\;SN}-n_{c,\;SC}=0$$ (4)

$$n_{c,\;\;SN}-n_{c,\;M}=0$$ (5)

$$n_{c,\;SC}-n_{c,\;M}=0$$ (6)

where *n* is the projected number in spreadsheet cell *c* for a given model (*SN*: Single Name, *SC*: Single Cell, *M*: Matrix). This process was performed separately for each model revision (Re-Entry into Care, Guideline-based ART, and Universal ART) and is shown in tabs Diff (SN-SC), Diff (SN-Matrix), and Diff (SC-Matrix) in supplementary files S2 File, S4 File, and S6 File.

We classified and characterized unintentional errors identified in Eqs 1–3. Unintentional errors were classified as due to: incorrect cell names, incorrect cell references, incorrect range(s) in a formula, incorrectly copied formulae, overwritten formulae, and misuse of built-in functions [29]. For each revision, we also examined: number, type, and location of errors; time required to implement each revision and correct errors; and error rate—the number of errors per total time spent implementing revisions and corrections. Employing the “original sin” rule, we counted errors only in the original cell where the error occurred [30]. However, we also tracked propagated errors, which were repeated with incorrectly copied formulae or when original errors resulted in errors in dependent cells.

To examine the impact of unintentional errors, we compared projections for models with versus without unintentional errors. We first identified our gold standard comparator, which was the corrected model fulfilling Eqs 4–6. For a given spreadsheet cell in each model version, we then calculated the percentage difference between the revised (but incorrect) model version and the subsequent revised, gold-standard model that was error-free to our knowledge [10] (Eqs 7–9):

$$\frac{n_{c,\;SN,\;errors}-n_{c,\;SN,\;no\;errors}}{n_{c,\;SN,\;no\;errors}}\times 100={\%\;\mathrm{difference}}_{c,\;\;SN}$$ (7)

$$\frac{n_{c,\;SC,\;errors}-n_{c,\;SC,\;no\;errors}}{n_{c,\;SC,\;no\;errors}}\times 100={\%\;\mathrm{difference}}_{c,\;SC}$$ (8)

$$\frac{n_{c,\;M,\;errors}-n_{c,\;M,\;no\;errors}}{n_{c,\;M,\;no\;errors}}\times 100={\%\;\mathrm{difference}}_{c,\;M}$$ (9)

where *n* is the projected number in spreadsheet cell *c* for a given model (SN: Single Name, SC: Single Cell, or M: Matrix) that either has unintentional errors (denoted as *errors*) or has no known unintentional errors (denoted as *no errors*). These calculations are shown in detail in S7 File. Our threshold for error seriousness was 5%, a threshold for an error to be considered material [31].

We also assessed the impact of errors for outcomes along the HIV care continuum (i.e., total number HIV-infected, diagnosed and linked to care, receiving ART, retained in care prior to ART and retained in care when receiving ART), a policy-relevant lens for evaluating HIV programs [32, 33]. To do so, we calculated the percentage difference in the number in each step along the care continuum by 2023 between model versions with and without unintentional errors, similar to Eqs 7–9. Model versions with and without unintentional errors for each of the three revisions are available as supporting information in S1–S7 Files.

## Results

### Types of unintentional errors and unintentional error rate

Across all revisions and versions, 58 original unintentional errors occurred, with most due to incorrect cell references (69%) or names (28%) (Table 1). The 58 original unintentional errors occurred in a total of 2,208 new or revised model spreadsheet cells (736 changed cells / model version x 3 model versions = 2,208), with approximately 3% of changed model cells having an original unintentional error. The number of original unintentional errors per changed cell was similar across model versions (2%, Single Name; 3%, Single Cell; 2%, Matrix), although was as high as 8% for the Universal model revision. Across all model versions and revisions, we identified 4,331 propagated errors arising from the original unintentional errors, resulting in an overall unintentional error rate of 28% ([58 original errors + 4,331 propagated errors] / [5,140 total model cells / model version x 3 model versions] * 100% = 28%). The most original unintentional errors (24 errors) occurred in Single Cell, which also required the most implementation time (8.9 hours; 2.8 original unintentional errors/hour). While the least time (4.3 hours) was spent revising and correcting Single Name, it had the most propagated unintentional errors and highest unintentional error rate (2173 propagated unintentional errors; 4.2 original unintentional errors/hour). Matrix had the fewest original and propagated errors and the lowest error rate.

**Table 1:** Number, type, and rates of unintentional errors, by model version and revision.

Version	Revision	Unintentional errors (number)				Model cells		Unintentional errors / model cell (%)		Time (hours)			Unintentional error rate (number / hour)	
		Original	Type	Propagated	Total	New or revised model cells	Total model cells	Original errors / changed cell	Total errors* / model cell	Revise model	Identify, correct errors	Total	Original†	Propagated‡
Single Name	Re-entry	1	Incorrect cell name (1)	146	147	76	1,360	1%	11%	0.7	<0.1	0.7	1.5	213.7
	Universal	7	Incorrect cell references (4); Incorrect cell name (3)	851	858	220	1,640	3%	52%	0.9	0.6	1.5	4.8	580.2
	Guideline	10	Incorrect cell reference (7); Incorrect cell name (3)	1178	1188	440	2,140	2%	56%	1.6	0.6	2.2	4.7	547.9
	*Subtotal*:	*18*		*2173*	*2193*	*736*	*5*,*140*	*2%*	*43%*			*4*.*3*	*4*.*2*	*505*.*8*
Single Cell	Re-entry	0	—	—	0	76	1,360	0%	0%	0.8	—	0.8	—	—
	Universal	17	Incorrect cell references (17)	841	858	220	1,640	8%	52%	1.1	3.3	4.4	3.8	190.4
	Guideline	7	Incorrect cell reference (6); Logic (1)	752	759	440	2,140	2%	35%	3.1	0.6	3.7	1.9	204.2
	*Subtotal*:	*24*		*1593*	*1617*	*736*	*5*,*140*	*3%*	*31%*			*8*.*9*	*2*.*8*	*179*.*0*
Matrix	Re-entry	0	—	—	0	76	1,360	0%	0%	0.7	—	0.7	—	—
	Universal	7	Incorrect cell reference (6); Typographical error (1)	409	416	220	1,640	3%	25%	1.8	0.7	2.4	2.9	168.1
	Guideline	9	Incorrect cell name (9)	154	163	440	2,140	2%	8%	3.1	1.1	4.2	2.1	36.4
	*Subtotal*:	*16*		*563*	*579*	*736*	*5*,*140*	*2%*	*11%*			*7*.*3*	*2*.*2*	*76*.*9*

* Total errors are defined as the sum of original and propagated errors.

† Original error rate: original unintentional errors (column 3) per total time spent revising the model and identifying and correcting unintentional errors (column 12).

‡ Propagated error rate: propagated unintentional errors (column 5) per total time spent revising the model and identifying and correcting unintentional errors (column 12).

### Impact of unintentional errors on model output

**Fig 3:**
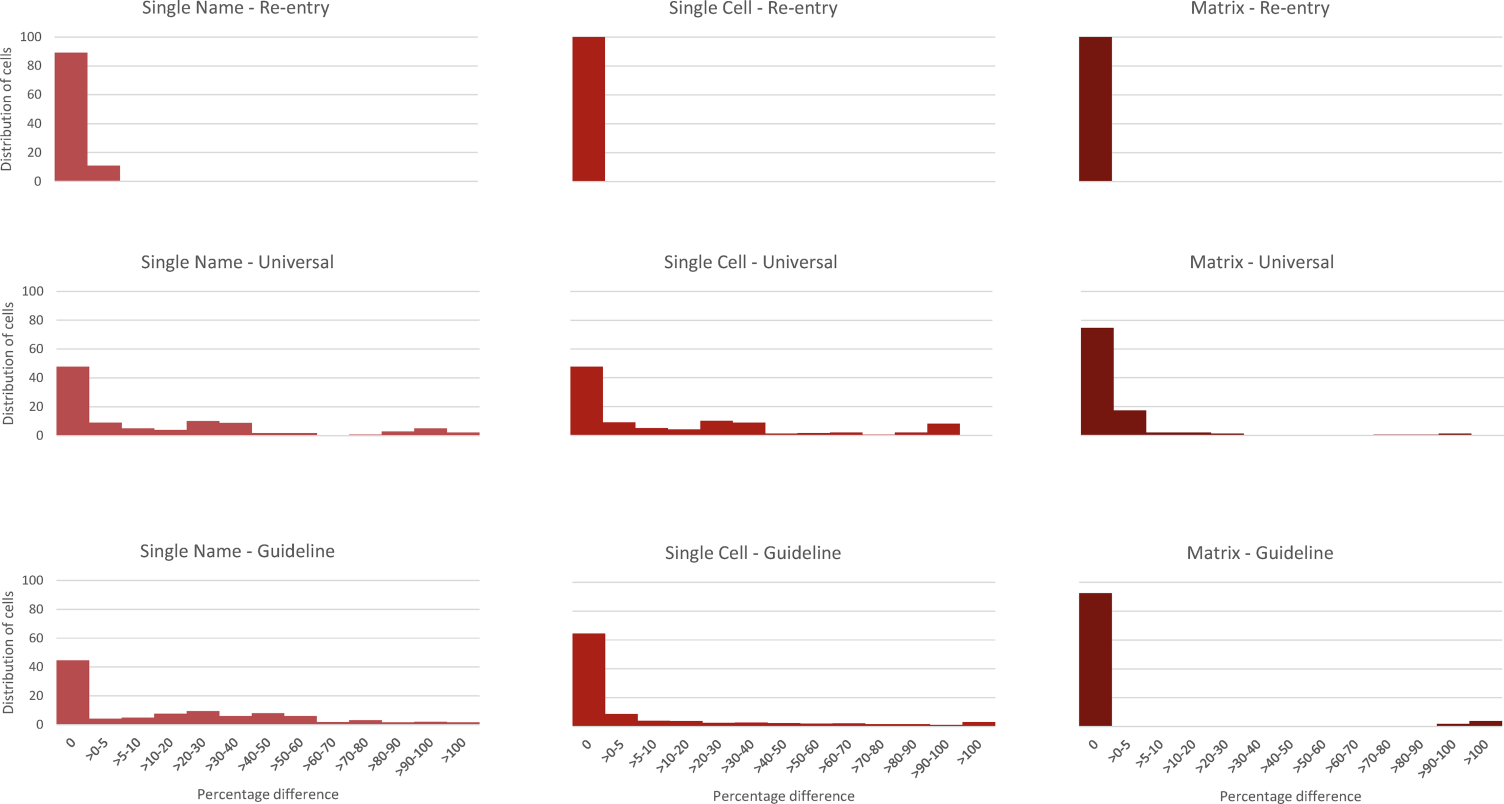
Distribution of the percentage difference in model output due to implementation errors, by model version and revision. This figure shows the distribution of the size of unintentional errors for each cell in the spreadsheet model. The horizontal axis shows the percentage difference in model projections for models with unintentional errors compared to the revised, gold-standard model without unintentional errors. The vertical axis shows the percentage of spreadsheet cells. Panels are organized from least complex model revision to most complex (top to bottom). A percentage difference of 0% indicates no unintentional error. No unintentional errors occur in the Re-entry revision of the Single Cell–Re-entry model version. However, nearly half of cells in the more complex Universal revision of the Single Cell model version produced a >5% difference between model projections with versus without unintentional errors.

We examined the impact of unintentional errors on model output (Table 2, Fig 3). Across all models and revision, 22% of model spreadsheet cells had model output differences of >5%, the threshold for an error to be considered material (3,373 material unintentional errors / [5,140 total model cells / model version x 3 model versions] * 100% = 22%) [31]. However, there was wide variation across model versions and revisions in the percentage of cells with material errors, ranging from 0% to 51% (Single Name), 0% to 43% (Single Cell), and 0% to 8% (Matrix). Some of the differences in model output arising from material unintentional errors were substantial: Overall, 6% of spreadsheet cells with material unintentional errors more than doubled model output across all model versions and revisions (Summary Table tab in S7 File). However, the greatest model output differences generally occurred in the more complex revision (e.g., Guideline-based ART) for the Single Name and Single Cell versions. At times, there were major differences in model output arising from unintentional errors. For example, in the Matrix model version (Guideline-based ART revision), 11 spreadsheet cells, or approximately 7% of spreadsheet cells with material unintentional errors, increased model output for a particular cell by more than eight times. Detailed information on the magnitude of error impact, by model and revision, is available in S7 File; for example, in the Re-entry, SN tab, Panel 1 shows the model output with unintentional errors, Panel 2 shows the model output with errors corrected, Panel 3 shows the percentage difference between the uncorrected and corrected models, and Panel 4 provides a summary of error magnitude.

**Table 2:** Frequency and percentage of error type.

	Frequency (percentage)† of spreadsheet cells																	
	Single Name						Single Cell						Matrix					
Type of error*	Re-entry		Universal		Guideline		Re-entry		Universal		Guideline		Re-entry		Universal		Guideline	
No error	1,213	(89)	782	(48)	952	(44)	1,360	(100)	782	(48)	1,381	(65)	1,360	(100)	1,224	(75)	1,977	(92)
Non-material error	147	(11)	150	(9)	91	(4)	0	(0)	147	(9)	187	(9)	0	(0)	285	(17)	9	(0)
Material error	0	(0)	708	(43)	1097	(51)	0	(0)	711	(43)	572	(27)	0	(0)	131	(8)	154	(7)

*A non-material error is defined as an error for which the percentage difference is ≤5% for model output in a given spreadsheet cell from model with errors versus the model without known errors. Errors with >5% difference for model output in a given spreadsheet cell were considered material errors.

† The total number of spreadsheet cells for the Re-entry revision is 1,360, for the Universal revision is 1,640, and for the Guideline revision is 2,140. Shown are column percentages for each revision in each model version. The overall percentage of spreadsheet cells with errors reported in the text is based on the sum of all errors or all material errors divided by the sum of all spreadsheet cells (15,420). Column percentages may not add to 100% due to rounding.

**Fig 4:**
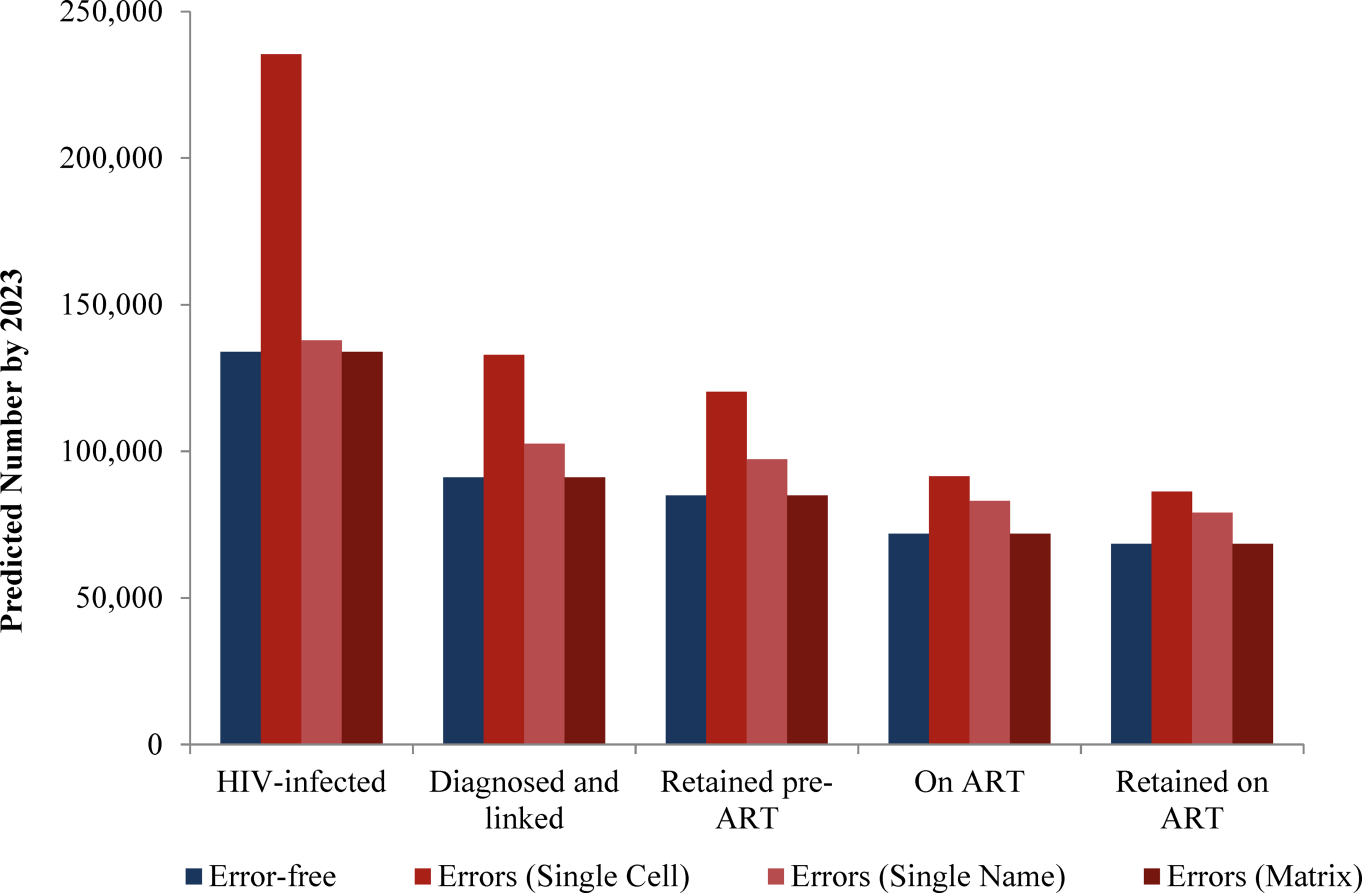
Impact of unintentional errors on model projections along the HIV care continuum. This figure shows the impact of unintentional errors on policy-relevant model projections for a complex model revision (Guideline-based ART). On the horizontal axis are five key steps along the HIV care continuum, while on the vertical axis are the projected estimates of the number in each step along the care continuum by 2023. Projections made by the error-free model are shown in blue, while model revisions with unintentional errors are shown in different shades of red, by model version (■ for Single Cell, ■ for Single Name, and ■ for Matrix).

In assessing the impact of unintentional errors on key steps along the HIV care continuum, we found wide variation in the percentage difference in model output for versions with and without unintentional errors, with more complex revisions generally resulting in larger projection differences. For example, differences in model output for less complex revisions ranged from 0% (Re‐entry) to –17% (Universal ART) across all model versions. The most complex revision implemented, Guideline-based ART, yielded projection differences ranging from 3% to +16% (Single Name), +26% to +76% (Single Cell), and 0% (Matrix) across the HIV care continuum (Fig 4).

## Discussion

Across all model versions, this methodologic case study analysis finds that implementation of model revisions generally results in unintentional errors. This demonstration also suggests that at times, unintentional errors can result in substantial policy implications, with near doubling of projections along one step in the HIV care continuum. While our demonstration cannot assess the extent to which unintentional errors may be due to random variability or propensity for larger errors in some model types, our results do illustrate the range of possible outcomes that might occur in the real world: unintentional errors may not materially affect projections, or they could change a study’s policy conclusions.

While there is no ‘best’ model structure, using multiple versions of the same model to validate results may be one way, in additional to traditional debugging approaches, to reduce unintentional errors in spreadsheet models that may be used to inform policy decisions. Our study corroborates recent findings emphasizing the importance of cross-model validation and use of multiple model versions to identify implementation errors and assess model assumptions [34]. While this process may be time consuming and potentially expensive, it is crucial for identifying unintentional errors. This approach can be relatively easily and routinely implemented for simple deterministic model structures with analytical solutions, as well as for more complex model structures (e.g., Monte Carlo simulation) by using fixed seeds for the generation of random numbers.

This approach could be especially valuable in settings with limited resources and technical capacity. Analysts with limited training are especially in need of simple, validated ways to verify model outputs in simple forecasting models they may have developed to inform local policy. High-quality evidence, including systematic and thorough model validation efforts, thus becomes a critical step not only in model implementation but in allocating scarce resources [35]. Given the potential for this method of error-checking to prevent policy mistakes, decision makers in resource-limited settings may find this approach of parallel model comparisons to be worth the resource investment.

This study’s findings contribute to an increasing literature on systems science model performance assessment, including error identification, in the health context. Best practice guidelines highlight the importance of debugging (i.e., error elimination), transparency, and broader model performance assessment processes [4, 36], as well as the importance of reporting these methods (e.g., see the CHEERS checklist [37]). Much of the existing literature addresses specific model validation and calibration approaches [38–45] or focuses on demonstrating adequate model performance for existing disease-specific models [34, 46–52]. Emerging work by Caro has introduced an approach to integrate different decision analytic modeling approaches both to improve usability of these methods for decision makers and to increase model transparency [53]. A much smaller health-related literature addresses a key domain of model performance—error identification—and focuses mainly on describing error taxonomy and current approaches to error identification in spreadsheet models. Recent work by Chilcott and colleagues describes errors in health technology assessment models and provides qualitative insights into defining modeling errors, avoiding these errors, and identifying them [7]. Tappenden and Chilcott extend this work by developing a formal taxonomy of errors, including those related to model implementation, that can undermine model credibility [28].

The current analysis thus extends existing knowledge in several key ways: First, we introduce an additional approach, beyond standard error identification techniques, to identify unintentional errors. Because standard debugging techniques are well-suited for identifying errors that can be identified in extreme conditions (e.g., extreme value analysis, negative output tests), the current method offers an additional approach for identifying material errors that may otherwise go unnoticed. Indeed, this is among the first studies in the health modeling literature that contributes empirical evidence on the frequency and impact of unintentional errors. Second, we explicitly adopt a material error threshold with which to evaluate potential implementation errors, thresholds that are largely lacking in the health-related literature. While some existing work has referenced error thresholds in the context of assessing internal validity [13, 54] or performing cross-model validation [34], we are among the first, to our knowledge, to explicitly examine unintentional errors within a defined threshold. Finally, in an illustrative example, we highlight the potential impact of failing to identify material unintentional errors on policy decisions. Although such evidence exists outside the health sector [55–57], evidence quantifying how unintentional errors could influence health decision making is scant.

Our work has several limitations. First, we used a model representing clinical engagement for a single disease and the model’s simple conceptual structure may not be applicable to all clinical policy questions. Second, mimicking constraints similar to resource-limited conditions, we conducted a methodologic case study analysis rather than a more rigorous experimental design, which suggests that our findings should be interpreted with caution. Specifically, one individual implemented model revisions and only one time, which did not allow us to assess measures of central tendency or random variability in our projections. Further, the single programmer implementing the model versions and revisions was knowledgeable about the model, which may have served to underestimate the number, and therefore the impact, of potential unintentional errors as well as the total time taken to implement model revisions, identify unintentional errors, and correct them. Similarly, an approach relying on a single programmer to implement multiple model versions (versus multiple programmers implementing a single model version [4]) may have resulted in repeated programming errors, thereby overestimating unintentional errors and their impact. However, despite these limitations, our findings suggest that unintentional errors occur when implementing spreadsheet models, that they can be identified, and that they could have significant policy implications. This type of timely and practical solution for identifying unintentional errors is crucial for resource-limited settings that may rely on internal spreadsheet-based models to inform their policy decisions, such as health departments or Ministries of Health.

Third, we did not have available a single gold-standard, error-free model with which to identify implementation errors and make model projection comparisons. Because each model revision was made concurrently across model versions, we instead adopted an alternative approach: identification and correction of unintentional errors for each model version, resulting in an error-free model to our knowledge, which could be used as a comparator to its corresponding model with errors. We chose this approach to more thoroughly test the model’s behavior, and future work on comparative approaches for unintentional error identification is warranted. We also did not compare projections from a parallel model implemented in a different software platform, an approach that may provide additional opportunities to identify unintentional errors [7]. Regardless of the software used for model implementation, however, findings from this work emphasize the importance of internal model validation as a means to confirm model implementation. In addition to the error identification approach described in the current analysis, a more comprehensive systematic, iterative model performance assessment process is also critical, particularly one that examines both internal and external validity [13]. Yet even comprehensive model validation efforts cannot ensure that a model reflects reality. Finally, while we found substantial differences in model projections using model versions with unintentional errors versus without, it is unclear whether a given policy decision would have changed as a result of unintentional errors as we lack additional economic cost or budget data as well as empirical data on how decision makers use model projections to inform policy.

## Conclusions

In this research, we provide evidence that developing parallel model versions aids in identifying and resolving unintentional errors when revising spreadsheet models. This work suggests that standard error identification techniques may not identify all spreadsheet model errors and that unintentional errors can have a profound impact on model projections. Standard debugging techniques and modeling best practices are always recommended, but in some contexts, limited resources may preclude the use of standard but resource-intensive error identification techniques. As systems science approaches are scaled and begin to reach a variety of consumers, including decision makers who are well-positioned to respond to model findings, care should be taken to identify unintentional errors and promote reliable model projections. Implementation of parallel model versions during model development and revision is one means to do so.

## Supporting information

S1 File Re-entry_errors. This file contains the three model versions for the Re-entry into Care model revision, before identification of unintentional implementation errors.
(XLSX)

S2 File Re-entry_errors_corrected. This file contains the three model versions for the Re-entry into Care model revision, after identification and correction of unintentional implementation errors.
(XLSX)

S3 File Universal ART_errors. This file contains the three model versions for the Universal ART model revision, before identification of unintentional implementation errors.
(XLSX)

S4 File Universal ART_errors_corrected. This file contains the three model versions for the Universal ART model revision, after identification and correction of unintentional implementation errors.
(XLSX)

S5 File Guideline-based ART_errors. This file contains the three model versions for the Guideline-based ART model revision, before identification of unintentional implementation errors.
(XLSX)

S6 File Guideline-based ART_errors_corrected. This file contains the three model versions for the Guideline-based ART model revision, after identification and correction of unintentional implementation errors.
(XLSX)

S7 File Magnitude of unintentional errors. This file contains model output for a given model revision and version *with* unintentional errors, model output for a given model revision and version *without* unintentional errors, calculation of the error magnitude (defined as the percentage difference in model output for each spreadsheet cell for models with versus without unintentional errors), and the distribution of error magnitude.
(XLSX)
